# Passive Droplet Generation in T-Junction Microchannel: Experiments and Lattice Boltzmann Simulations

**DOI:** 10.3390/mi16091011

**Published:** 2025-08-31

**Authors:** Xiang Li, Weiran Wu, Zhiqiang Dong, Yiming Wang, Peng Yu

**Affiliations:** 1 Guangdong Provincial Key Laboratory of Turbulence Research and Applications, Department of Mechanics and Aerospace Engineering, Southern University of Science and Technology, Shenzhen 518055, China; lix8@sustech.edu.cn (X.L.);; 2 Advanced Institute for Ocean Research, Southern University of Science and Technology, Shenzhen 518055, China; 3 Shenzhen Key Laboratory of Complex Aerospace Flows, Southern University of Science and Technology, Shenzhen 518055, China; 4 Center for Complex Flows and Soft Matter Research, Southern University of Science and Technology, Shenzhen 518055, China

**Keywords:** microfluidic technology, microdroplet generation, multiphase flows, micro-PIV, lattice Boltzmann method, diffuse interface method

## Abstract

The present study investigates passive microdroplet generation in a T-junction microchannel using microscopic observations, microscale particle image velocimetry (Micro-PIV) visualization, and lattice Boltzmann simulations. The key flow regimes, i.e., dripping, threading, and parallel flow, are characterized by analyzing the balance between hydrodynamic forces and surface tension, revealing the critical role of the flow rate ratio of the continuous to dispersed fluids in regime transitions. Micro-PIV visualizes velocity fields and vortex structures during droplet formation, while a lattice Boltzmann model with wetting boundary conditions captures interface deformation and flow dynamics, showing good agreement with experiments in the dripping and threading regimes but discrepancies in the parallel flow regime due to neglected surface roughness. The present experimental results highlight non-monotonic trends in the maximum head interface and breakup positions of the dispersed fluid under various flow rates, reflecting the competition between the squeezing and shearing forces of the continuous fluid and the hydrodynamic and surface tension forces of the dispersed fluid. Quantitative analysis shows that the droplet size increases with the flow rate of continuous fluid but decreases with the flow rate of dispersed fluid, while generation frequency rises monotonically with the flow rate of dispersed fluid. The dimensionless droplet length correlates with the flow rate ratio, enabling tunable control over droplet size and flow regimes. This work enhances understanding of T-junction microdroplet generation mechanisms, offering insights for applications in precision biology, material fabrication, and drug delivery.

## 1. Introduction

Microscale multiphase flows, particularly droplet-based microfluids, have garnered substantial attention for their complex interfacial behaviors and versatile capabilities in high-throughput biological assays [1], functional material fabrication [2], and emulsion production [3]. While this technology has enabled diverse applications in these fields, the microscale multiphase flow mechanisms governing microdroplet generation remain insufficiently understood, limiting the rational design of high-performance microfluidic devices [4,5,6,7,8,9]. Currently, microdroplet generation methods are primarily classified into two categories: active methods driven by external fields [5,8] and passive methods relying on shear effects [6,9]. Unlike active methods that depend on external fields (e.g., electric, magnetic, or acoustic actuation), passive approaches manipulate the immiscible two-phase fluids, i.e., the continuous phase and dispersed phase, to squeeze and shear each other, thereby generating monodisperse microdroplets through hydrodynamic interactions without any additional external fields [4]. Depending on geometric configuration and flow properties, passive methods can be categorized into three distinct types, i.e., co-flow microchannels [10,11], cross-junction microchannels [12,13,14,15,16], and T-junction microchannels [17,18,19,20,21,22,23].

Recently, cross-junction and T-junction microchannels have received considerable attention due to their easy fabrication, simple operation, flexible parameter adjustment, and low cost. Cubaud et al. [12] experimentally investigated the microdroplet generation process in a cross-junction microchannel and found that the flow patterns are shifted from dripping to jetting by adjusting the flow rate of the continuous phase. Yu et al. [13] developed a three-dimensional numerical model based on the Volume of Fluid (VOF) method and proved that the flow pattern shifts result from not only the flow rate, but also complex structure–fluid interactions. Then, Wu et al. [14] observed a slug flow between dripping and jetting and established a scaling law based on the flow rate and capillary number. Ramji et al. [15] further investigated the mass transfer phenomenon during the slug flow in a cross-junction using the VOF method. Besides the mechanisms for the flow pattern shift, Liu et al. [16] focused on the liquid bridge breaking process at different width ratios of microchannels. From the above significant literature, the flow mechanisms for cross-junction microchannels have been well studied, but those for T-junction microchannels remain insufficiently understood because of their asymmetric nature.

In the realm of experimental investigations, Thorsen et al. [17] made a pioneering contribution by designing a T-junction microchannel to generate monodisperse microdroplets dominated by the viscous effect. This microchannel was fabricated by pouring acrylated urethane onto a silicon wafer-based mold. Even though the geometric accuracy of the microchannel is not good enough, this work still provides a possibility for microdroplet generation by T-junction microchannels. Garstecki et al. [18] statistically analyzed the droplet and bubble sizes generated in the T-junction but neglected the fluid mechanics and interface interaction. Jena et al. [19] investigated the geometric effect of microchannels on the droplet generation process. Another approach is to insert a capillary tube into a straight microchannel directly, benefiting from low cost and easy fabrication processes [20,21]. Dangla et al. [22] designed a sharp and thin nozzle to assist the breaking process of the dispersed phase, but its underlying mechanisms remain unrevealed. Recently, Pang et al. [23] filled the research gap on this structure and focused on the differential pressure force of droplets after they are generated from the nozzle. In the past, research was mainly focused on the effect of fluid properties and geometric structures on the microdroplet generation process in T-junction microchannels [9,24,25,26,27,28,29]. Recently, Shen et al. [21] employed microscale particle image velocimetry (Micro-PIV) to observe the vortex structure inside the microdroplet. Kovalev et al. [30] observed the velocity field distribution of parallel flow in T-junction microchannels by Micro-PIV and investigated the stability of the interface between silicone oil and water.

For the numerical simulations, commercial software, such as CFX, Fluent, and COMSOL Multiphysics, is used to simulate the microdroplet generation process in different microchannels, but most of these simulations have neglected the effects of the wetting boundary and fluid–structure interaction [31,32,33,34,35]. Chaghagolani et al. [35] investigated the droplet sorting process in a Y-junction microchannel, and the fluid–structure interaction was simulated by coupling the boundary element method with COMSOL Multiphysics. Li et al. [36] employed the VOF method to track the interface behavior and investigated the elongation rate of the dispersed fluid in T-junction microchannels. Another approach is to capture the interface deformation by solving the Cahn–Hilliard equation and describe the flow field by solving the Navier–Stokes equations [37,38,39]. In recent years, the lattice Boltzmann method has emerged as a powerful computational tool, enabling the simultaneous calculation of the Navier–Stokes equations and the Cahn–Hilliard equations [40,41,42,43,44,45,46]. It has proven particularly effective in accurately simulating complex multiphase flows, providing valuable insights into the dynamic behavior of different phases within microfluidic systems [39,47,48,49,50].

Unlike cross-junction microchannels, for which the effects of flow parameters on controlling the flow patterns have been well studied, the related phenomenon in T-junction microchannels has received relatively less attention. Thus, the present study aims to investigate the passive microdroplet generation in a T-junction microchannel by experimental observation and lattice Boltzmann simulations. Here, the impact of key flow properties, i.e., the flow rate ratio of the continuous and dispersed fluids, on the breakup process of dispersed fluid and droplet motion during the microdroplet generation is meticulously observed using a microscope with a high-speed camera. Additionally, the Micro-PIV is applied as an auxiliary tool to assist in the general observation of the velocity field, providing supplementary insights into the flow dynamics. Moreover, a mass-conserving multiphase lattice Boltzmann method [51,52,53] is employed to simulate the breakup process of dispersed fluid in the T-junction microchannel. For precisely simulating the wettability of the microchannel wall, the wetting boundary condition [39,54] is imposed.

The remainder of this article is organized as follows. In Section 2, the fabrication of the T-junction microchannel and the experiment setup are first introduced, and the macroscopic governing equations and their corresponding lattice Boltzmann model are then presented. In Section 3, the microdroplet generation process in the T-junction microchannel is observed through a microscope and CCD camera, and flow properties are characterized using Micro-PIV experiments and lattice Boltzmann simulations. Finally, the conclusions are drawn in Section 4.

## 2. Experimental and Numerical Methodology

### 2.1. Fabrication of the T-Junction Microchannel

**Figure 1:**
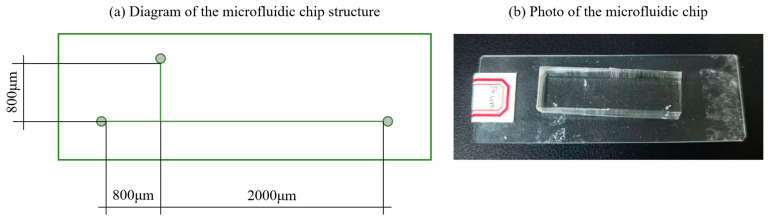
Microfluidic chip: (**a**) diagram and (**b**) photo.

Microfluidic chips based on T-junction microchannels have been successfully fabricated in past studies [17,28], though their microchannel sizes typically ranged from 200 to 500 μm in width and depth. To investigate the underlying mechanisms of microscale droplet generation, a T-junction microchannel with a width of 50 μm and depth of 60 μm is developed in the present study, which is shown in Figure 1. The lengths of both inlet channels for the continuous and dispersed phases are 800 μm, ensuring sufficient distance for the flow to fully develop. The downstream channel of the T-junction extends 2000 μm in length, which reduces the potential disturbance from the outlet flow.

A standard soft lithography process is performed to manufacture the pattern of the above design on a silicon wafer. The SU8 photoresist (2025, MicroChem, Austin, TX, USA) is spin-coated on a 4-inch silicon wafer at 1500 rpm, followed by a two-step thermal pre-treatment: it is first heated to 65 °C for 5 min, then heated to 95 °C and baked for 10 min. Before photolithography, it is naturally cooled to below 50 °C. The treated silicon wafer is exposed to UV light for 4 s in lithography equipment (UV-KUB2, KLOE, Saint-Mathieu-de-Tréviers, France), followed by 2–4 cycles of development and washing. For these steps, SU8 developer (MicroChem, USA) and isopropyl alcohol (IPA, 99.5%, Macklin, Shanghai, China) are used as the development and washing agents, respectively. Following air drying to remove the residual IPA on the surface of the silicon wafer, the mold for the T-junction microchannel is prepared. To fabricate microfluidic chips, 30.0 g of Polydimethylsiloxane (PDMS) pre-polymer and 3.0 g of cross-linker (SYLGARD 184 Silicone Elastomer Kit, Dow, Midland, MI, USA) are mixed by stirring. After defoaming, the PDMS elastomer is carefully poured onto the silicon wafer to fill the mold. The PDMS elastomer is molded by heating at 85 °C for 1 h. The microfluidic chips are prepared through cutting, punching, and bonding onto a glass slide. It is worth noting that although the designed width of the microchannel is 50 μm, the measured width is 51.44 μm, indicating a relative deviation of less than the lithography line width of 2 μm. The contact angle of the present PDMS surfaces is 109.57°, which is measured by a contact angle meter (OCA15EC, Dataphysics Instruments GmbH, Filderstadt, Germany).

### 2.2. Experimental Setup

**Figure 2:**
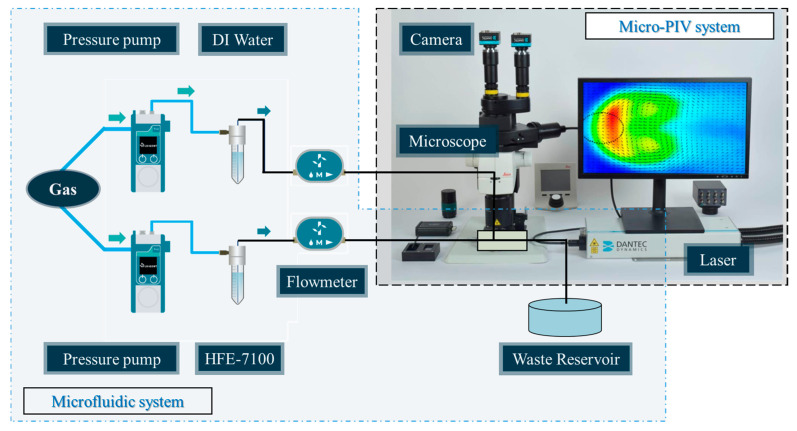
Scheme of the experimental setup.

The experimental setup includes microfluidic and Micro-PIV systems, and its arrangement is shown in Figure 2. The deionized water (DI water, Macklin) and hydrofluoroether (HFE-7100, 3M, St. Paul, MN, USA) are used as the dispersed and continuous fluids, respectively, and their physical properties are given in Table 1. Moreover, the fluorescent tracing microparticles with a particle diameter of 2 μm and an excitation wavelength of 535 nm (FluoSpheres 535/575, Thermo Fisher Scientific, Waltham, MA, USA) are added to DI water to track the flow velocity in Micro-PIV experiments, and the particle concentration is 0.05% *w*/*v*. The microstructures with a relatively lower particle concentration have less effect on the viscoelastic properties and surface tension, which has been investigated in the previous work [55]. The immiscible two-phase fluids are driven into microfluidic chips using two independent pressure pumps (Flow EZ, Fluigent, Le Kremlin-Bicêtre, France), and the flow rate of each fluid is measured by two separate flowmeters (Flow UNIT-M, Fluigent, France). All the units of the flow rate are μL/min. The pressure pumps and flowmeters are controlled by OxyGEN Software (version 2.3.2, Fluigent, France).

To observe the passive droplet generation phenomenon in the T-junction microchannel, a microscope (BX53, Olympus, Tokyo, Japan) is employed. Subsequently, the process is captured and recorded using a high-speed camera (FASTCAM AX100, Photron, Tokyo, Japan) in conjunction with a laptop (Precision 7540, Dell, Austin, TX, USA). The images are captured at a resolution of 1024 × 512 and a frame rate of 4000 fps, which is conducive to the subsequent analysis of the droplet generation process. All experiments are performed at least 10 times under a room temperature of 20 (± 0.5) °C, which can guarantee the reliability of the present study.

Additionally, a Micro-PIV experiment is performed to investigate the velocity field of the dispersed fluid during the passive droplet generation process. A dual-pulse Nd:YAG laser (Nano S 65–15 PIV, Litron Lasers, Rugby, UK) with a repetition rate of 15 Hz and pulse energy of 65 mJ, emitting at a wavelength of 532 nm, is used to excite the fluorescent tracing microparticles in DI water. These particles subsequently emit fluorescence at a wavelength of 575 nm, and their fluorescence images are observed using a fluorescence stereo microscope (M165 FC, Leica, Wetzlar, Germany) and captured by a dual-frame CCD camera (FlowSense EO, IMPERX, Sofia, Bulgaria). To ensure the coordinated operation of these devices, the dual-pulse Nd:YAG laser and dual-frame CCD camera are controlled by a performance synchronizer (Dantec Dynamics, Skovlunde, Denmark). Furthermore, all the hardware operations and Micro-PIV analyses are carried out via DynamicStudio software (version 7.6, Dantec Dynamics, Denmark). To ensure the reliability of Micro-PIV experiments, the focal plane for data acquisition is set as the middle height of the microchannel.

### 2.3. Governing Equations and Lattice Boltzmann Model

The passive droplet generation process is modeled as an incompressible, immiscible two-phase flow governed by the Navier–Stokes and Cahn–Hillard equations (with a mass correction term *Q*), which are expressed as:

(1) $$\frac{\partial \rho }{\partial t}+\nabla \left(\rho u\right)=0,$$

(2) $$\frac{\partial \rho u}{\partial t}+\nabla \cdot \left(\rho uu\right)=-\nabla p+\nabla \left[\mu \cdot \left(\nabla u+{\left(\nabla u\right)}^{T}\right)\right]+F_{s},$$

(3) $$\frac{\partial C}{\partial t}+\nabla \cdot \left(Cu\right)=M\nabla^{2}\mu_{C}+Q,$$

with

(4) $$Q=\left\{\begin{array}{ll} \frac{1}{\left|\delta V\right|}\cdot \left(\frac{1}{\left|\delta \rho \right|}\frac{\partial m}{\partial t}-\int_{\Omega_{L}}M\nabla^{2}\mu_{C}dV\right), & 0<\xi <1,\\ 0, & elsewhere, \end{array}\right.$$

where *ρ*, **u**, *p*, and *μ* are the fluid density, velocity, pressure, and dynamic viscosity, respectively. The mobility *M* represents the diffusion rate of Equation (3), which introduces a mass deviation during the interface deformation. The mass correction term *Q* only takes effect on the interface [51,52,53], where *δV* is the volume unit of the interfacial layer, *δρ* is the density difference, and $\frac{\partial m}{\partial t}$ is the mass loss rate of the lighter phase. The surface force term **f***_s_* is calculated by

(5) $$f_{s}=\mu_{C}\nabla C,$$

where *C* is the order parameter; *μ_C_* is the chemical potential, which is given as

(6) $$\mu_{C}=2AC\left(C-1\right)\left(2C-1\right)-\kappa \nabla^{2}C,$$

where the coefficients *A* and *κ* are defined by the interfacial thickness *W* and the surface tension coefficient *σ* as

(7) $$A=\frac{12\sigma }{W},$$

(8) $$\kappa =\frac{3W\sigma }{2}.$$

To solve the governing equations, i.e., Equations (1)–(3), a double-distribution lattice Boltzmann method with a D2Q9 lattice velocity model is employed in this work, which is written as [51,52,53]

(9) $$f_{\alpha }\left(r+e_{\alpha }\delta t,t+\delta t\right)-f_{\alpha }\left(r,t\right)=-\frac{1}{\tau_{f}}\left(f_{\alpha }\left(r,t\right)-f_{\alpha }^{eq}\left(r,t\right)\right)+S_{\alpha }\delta t,$$

(10) $$g_{\alpha }\left(r+e_{\alpha }\delta t,t+\delta t\right)-g_{\alpha }\left(r,t\right)=-\frac{1}{\tau_{g}}\left(g_{\alpha }\left(r,t\right)-g_{\alpha }^{eq}\left(r,t\right)\right)+G_{\alpha }\delta t,$$

with

(11) $$S_{\alpha }=\left(1-\frac{1}{2\tau_{f}}\right)\frac{\left(e_{\alpha }-u\right)}{c_{s}^{2}}\cdot \left[\Gamma_{\alpha }\left(u\right)\left(F_{g}+F_{s}+F_{m}\right)-\nabla \rho c_{s}^{2}\left(\Gamma_{\alpha }\left(u\right)-\omega_{\alpha }\right)\right],$$

(12) $$G_{\alpha }=\left(1-\frac{1}{2\tau_{g}}\right)\omega_{\alpha }Q,$$

(13) $$\Gamma_{\alpha }\left(u\right)=\omega_{\alpha }\left[1+\frac{e_{\alpha }\cdot u}{c_{s}^{2}}+\frac{{\left(e_{\alpha }\cdot u\right)}^{2}}{2c_{s}^{4}}-\frac{{\left|u\right|}^{2}}{2c_{s}^{2}}\right],$$

(14) $$f_{\alpha }^{eq}\left(r,t\right)=\rho \Gamma_{\alpha }\left(u\right)=\omega_{\alpha }\rho \left[1+\left(\frac{e_{\alpha }\cdot u}{c_{s}^{2}}+\frac{{\left(e_{\alpha }\cdot u\right)}^{2}}{2c_{s}^{4}}-\frac{{\left|u\right|}^{2}}{2c_{s}^{2}}\right)\right],$$

(15) $$g_{\alpha }^{eq}=\left\{\begin{array}{cc} C-\mu_{C}M\left(1-\omega_{0}\right)/c_{s}^{2} & \alpha =0\\ \omega_{\alpha }\left(\mu_{C}M+Ce_{\alpha }\cdot u\right)/c_{s}^{2} & \alpha =1-8 \end{array}\right.,$$

where $f_{\alpha }\left(r,t\right)$ and $g_{\alpha }\left(r,t\right)$ denote the distribution functions of the hydrodynamics parameters and the order parameter, respectively, associated with the lattice velocity **e***_α_* and the weighting coefficient *ω_α_*. Correspondingly, $f_{\alpha }^{eq}\left(r,t\right)$ and $g_{\alpha }^{eq}\left(r,t\right)$ are their equilibrium distribution functions. *δt* is the time interval, *c_s_* is the speed of sound, and *S_α_* and *G_α_* are the source terms for the external force and the mass correction, respectively. The single relaxation parameters in Equations (9) and (10), i.e., *τ_f_* and *τ_g_*, can be defined by *μ* and *M* as

(16) $$\tau_{f}=\frac{\mu }{\rho c_{s}^{2}\delta t}+\frac{1}{2},$$

(17) $$\tau_{g}=\frac{M}{Q_{d}\delta t}+\frac{1}{2},$$

where *c_s_* and *Q_d_* are the speed of sound and the diffusion parameter to adjust the relaxation parameters, respectively. To simulate the wettability effect of the T-junction microchannel, a wetting boundary condition based on the geometric surface energy formulation proposed by Liang et al. [39] and Ding et al. [54] is imposed in the present work. The contact angle can be given as

(18) $$\tan\left(\frac{\pi }{2}-\theta \right)=\frac{-n\cdot \nabla C}{\left|\nabla C-\left(n\cdot \nabla C\right)n\right|},$$

where *θ* is the contact angle and **n** is the unit outward normal defined at the solid surface. For the convenience of numerical computation, applying the central finite difference scheme to discretize the above equation yields

(19) $$C_{i,0}=C_{i,2}+\tan\left(\frac{\pi }{2}-\theta \right)\left|C_{i+1,1}-C_{i-1,1}\right|.$$

where *C*_*i*,0_, *C*_*i*,1_, and *C*_*i*,2_ are the order parameters on the boundary nodes of the computational domain, the boundary nodes of the physical domain, and the fluid nodes, respectively.

## 3. Results and Discussion

### 3.1. Flow Regimes and Their Transition Mechanisms

**Figure 3:**
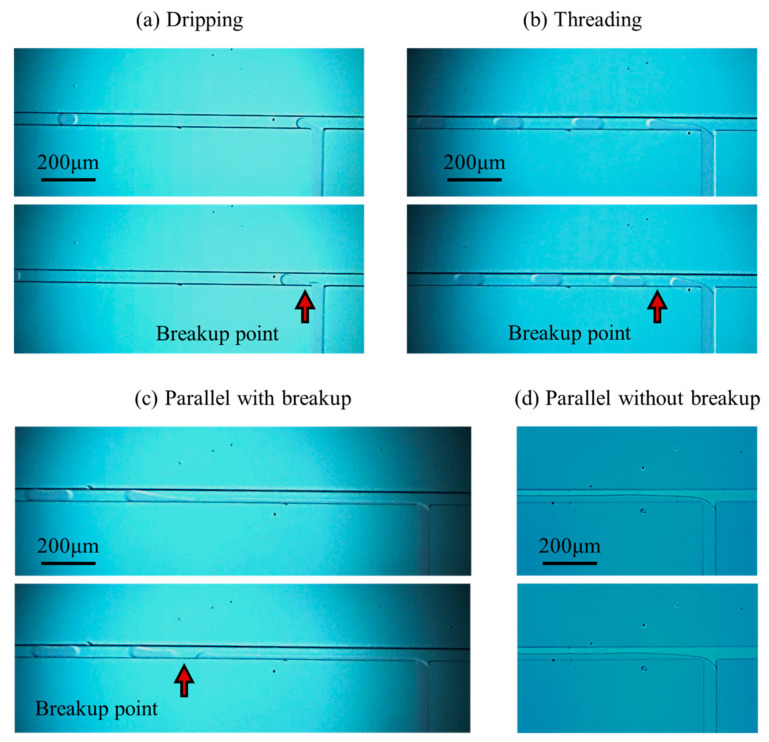
Flow regimes in the T-junction microchannel: (**a**) dripping regime, (**b**) threading regime, (**c**) parallel flow regime with breakup pattern, and (**d**) parallel flow regime without breakup pattern.

Generally, the flow regimes in T-junction microchannels are classified as dripping regime, threading regime, and parallel flow regime [25,27]. However, the microchannel width in the present work is smaller than that in past studies. Owing to the boundary layer effect, the interface of the dispersed fluid in the parallel flow regime is induced to break up, leading to the observation of two distinct flow patterns within this regime: a breakup pattern and a non-breakup pattern. The observed flow regimes and their corresponding patterns in the present experiment are illustrated in Figure 3.

In the dripping regime Figure 3a, the DI water completely occupies the microchannel of the dispersed fluid. The pressure of HFE-7100 adjacent to the DI water increases when the DI water occupies the T-junction, and then the HFE-7100 squeezes the DI water to break up into microdroplets. However, in the threading regime Figure 3b, a visible boundary between DI water and HFE-7100 is observed in the microchannel of the dispersed fluid. The leading edge of the DI water interface extends along the flow direction, and the microdroplets are generated under the squeezing and shearing effects of HFE-7100 on the DI water. In the parallel flow regime Figure 3c,d, a stable two-phase interface between DI water and HFE-7100 forms at relatively high flow rates of the dispersed and continuous phases. When the flow rate of dispersed fluid (*Q_D_*) is lower than that of continuous fluid (*Q_C_*), the thin liquid bridge of DI water breaks up into microdroplets under the combined effects of squeezing and shearing forces from HFE-7100 and the boundary layer effect of the microchannel wall, as shown in Figure 3c. When *Q_D_* > *Q_C_*, the liquid bridge thickens and will not break, as shown in Figure 3d.

**Figure 4:**
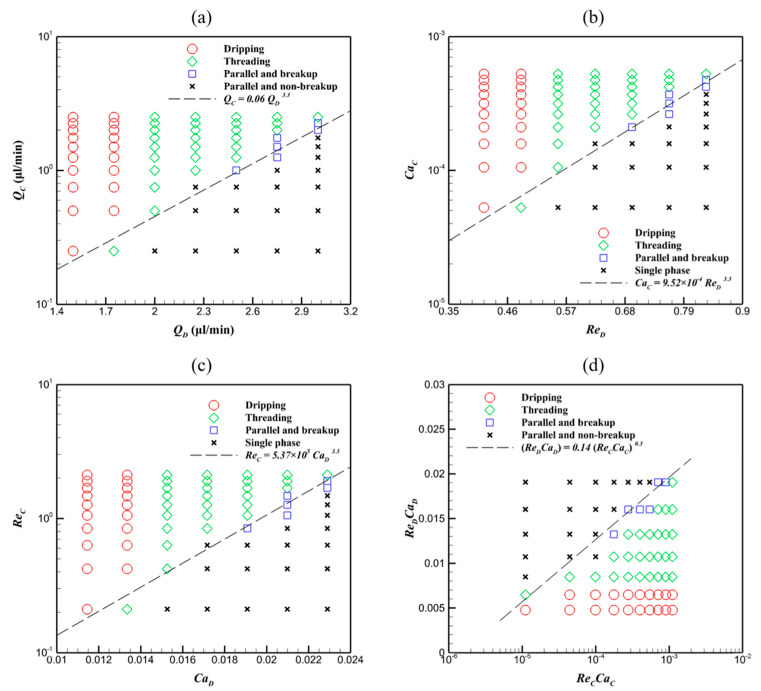
Flow regime map in the T-junction microchannel showing the correlation between (**a**) *Q_D_*–*Q_C_*, (**b**) *Re_D_*–*Ca_C_*, (**c**) *Ca_D_*–*Re_C_*, and (**d**) *Re_C_Ca_C_*–*Re_D_Ca_D_*.

Here, these flow regimes demonstrate that the key to generating microdroplets in the T-junction microchannel is the balance between the viscous, inertial, and surface tension forces. To describe the operation range of the flow regimes, the Reynolds number Re=QAρwcμ and capillary number $\mathrm{Ca}=\frac{Q}{A}\frac{\mu }{\sigma }$ are introduced, where $A=w_{c}\times d_{c}$ is the cross-sectional area of the microchannel and *w_c_* and *d_c_* are the width and depth of the microchannel, respectively. This indicates that the microdroplet generation process involves a competition between the Reynolds number and the capillary number. Although only HFE-7100 and DI water are used in the present work, varying flow rates enable the investigation of different flow regimes with different Reynolds and capillary numbers, which provides reference data for studies involving different geometric sizes or fluids with different properties. The flow regime map under different physical conditions is illustrated in Figure 4.

In Figure 4a, the flow regimes in the T-junction microchannel depend on the flow rate ratio between dispersed fluid (*Q_D_*) and continuous fluid (*Q_C_*). When $Q_{C}>0.06{Q_{D}}^{3.3}$, the hydrodynamic force of the continuous fluid drives and destabilizes the dispersed fluid interface, inducing breakup and microdroplet generation. When $Q_{C}<0.06{Q_{D}}^{3.3}$, the driving force of the continuous fluid is relatively smaller than that of the dispersed fluid, and the flow regime evolves into parallel flow. However, heightened *Q_D_* induces the interface instability of dispersed fluid, fragmenting the parallel flow into microdroplets, as shown in Figure 4c. In other words, the microdroplet generation process depends on the balance between Re*_D_* and Ca*_C_*, as well as Ca*_D_* and Re*_C_*, with the corresponding regime maps being shown in Figure 4b,c, respectively. Microdroplets are generated in the T-junction microchannel when either CaC>9.52×10−4ReD3.3 or ReC>5.37×105CaD3.3 is met; otherwise, microdroplet formation is not observed. The relation CaC>9.52×10−4ReD3.3 indicates that the surface tension effect of the continuous fluid is smaller than the hydrodynamic effect of the dispersed fluid, while the ReC>5.37×105CaD3.3 relation demonstrates that the hydrodynamic effect of the continuous fluid dominates over the surface tension effect of the dispersed fluid. To further reveal the combined effects of hydrodynamic force and surface tension force, the relation between Re*_C_*–Ca*_C_* and Re*_D_*–Ca*_D_* is illustrated in Figure 4d. It should be mentioned that Re×Ca=QA2ρwcσ is equivalent to the Weber number (We). When ReCCaC<0.14ReDCaD0.3, i.e., ${\mathrm{We}}_{C}<0.14{{\mathrm{We}}_{D}}^{0.3}$, the microdroplets are generated, which demonstrates that enhancing the surface tension of the continuous fluid or reducing the flow rate of the dispersed fluid, in conjunction with increasing the flow rate of the continuous fluid or lowering the surface tension of the dispersed fluid, promotes droplet formation.

Moreover, when the flow rate is below the range investigated in the present study, only single-phase flow exists in the microchannel. When the flow rate exceeds this range, only parallel flow is observed. Also, the transition between the two regimes may not be abrupt. While an exact critical flow rate for the transition might exist, it requires extremely precise flow rate control, which is not achievable in our current experiments. In numerical simulations, the thin layer in the parallel flow regime is hard to break.

### 3.2. Mechanism of Microdroplet Generation

To characterize the underlying flow properties, a series of lattice Boltzmann simulations and Micro-PIV experiments are performed. These techniques elucidate the hydrodynamic interactions and the interface dynamics during droplet generation, bridging the gap between flow regime transitions (Section 3.1) and the underlying physical mechanisms. In the lattice Boltzmann simulation, the relevant validations are presented in our past works [51,52], and all the simulations satisfy the mesh independence criteria. The width of the microchannel is set to 50 μm and discretized into 50 lattice units. The interface thickness and mobility of Cahn–Hilliard equations are set to 4 and 0.1, respectively. The contact angle in the present numerical simulation is set to 110°. No-slip boundary conditions are applied to the microchannel walls, while the inlet boundaries of DI water and HFE-7100 are defined using theoretical velocity profiles of Poiseuille flow to mimic the experimental flow rates. According to Figure 3a, the conditions of *Q_C_* = 1.0 μL/min, *Q_D_* = 1.5 μL/min, 2.0 μL/min, and 2.5 μL/min produce dripping, threading, and parallel with breakup regimes.

**Figure 5:**
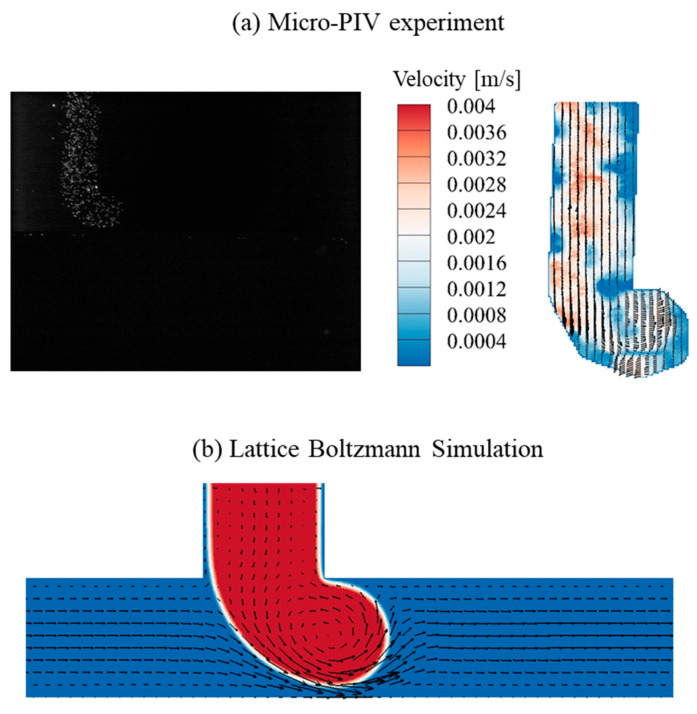
The interface and flow field obtained from the Micro-PIV experiment and the lattice Boltzmann simulation in the dripping regime (*Q_C_* = 1.0 μL/min, *Q_D_* = 1.5 μL/min).

For the dripping regime (*Q_C_* = 1.0 μL/min, *Q_D_* = 1.5 μL/min), the interface shape and flow field are characterized in Figure 5. The corresponding dimensionless numbers are *Re_C_* = 0.843, *Ca_C_* = 2.11 × 10^−4^, *Re_D_* = 0.416, and *Ca_D_* = 1.14 × 10^−2^, respectively. The interfacial motion and deformation of DI water are driven by its inherent driving pressure (controlled via dispersed-phase pressure pump) and skewed toward the outlet under the hydrodynamic squeezing effect of the continuous-phase HFE-7100, leading to the observed L-shaped deformation. Figure 5a shows the fluorescent image and velocity field in the dripping regime, with a vortex visualized at the center of the DI water head. This vortex structure is also observed in the lattice Boltzmann simulation, as shown in Figure 5b, which confirms the consistency between experimental observations and numerical results. The vortex structure generates a backflow to fill the microchannel of the dispersed fluid and resists the elongation toward the outlet, thereby maintaining the structural stability necessary for periodic droplet generation. As the DI water head blocks the HFE-7100 flow, pressure accumulates on the left of the DI water interface, after which the squeezing and shearing effect of HFE-7100 on the DI water interface results in droplet generation.

**Figure 6:**
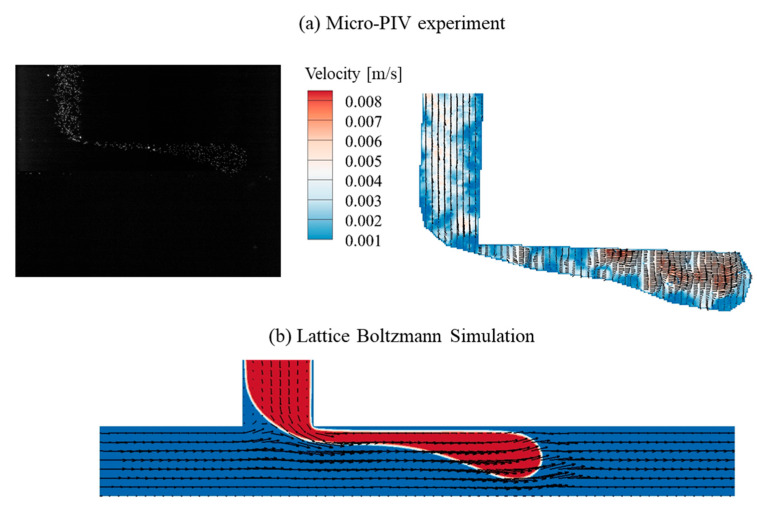
The interface and flow field obtained from the Micro-PIV experiment and the lattice Boltzmann simulation in the threading regime (*Q_C_* = 1.0 μL/min, *Q_D_* = 2.0 μL/min).

For the threading regime (*Q_C_* = 1.0 μL/min, *Q_D_* = 2.0 μL/min), the interface shape and flow field are characterized in Figure 6. The corresponding dimensionless numbers are *Re_C_* = 0.843, *Ca_C_* = 2.11 × 10^−4^, *Re_D_* = 0.555, and *Ca_D_* = 1.53 × 10^−2^, respectively. The DI water interface exhibits an L-shape, similar to that in the dripping regime, but the DI water head elongates under the enhanced hydrodynamic interaction at a higher flow rate of dispersed fluid. Both the Micro-PIV experiment and lattice Boltzmann simulation demonstrate that there is no obvious vortex in the threading regime. Compared with Figure 5a, a visible boundary between DI water and HFE-7100 is observed in the microchannel of the dispersed fluid. This boundary and the gooseneck-like thin layer of DI water are deformed by the balance between the hydrodynamic forces of HFE-7100 flow and the surface tension force of DI water, governing the transition from dripping to threading regimes. The DI water forms a head structure ahead of the gooseneck-like thin layer, which is subsequently squeezed and sheared by the HFE-7100 flow, leading to the generation of microdroplets.

**Figure 7:**
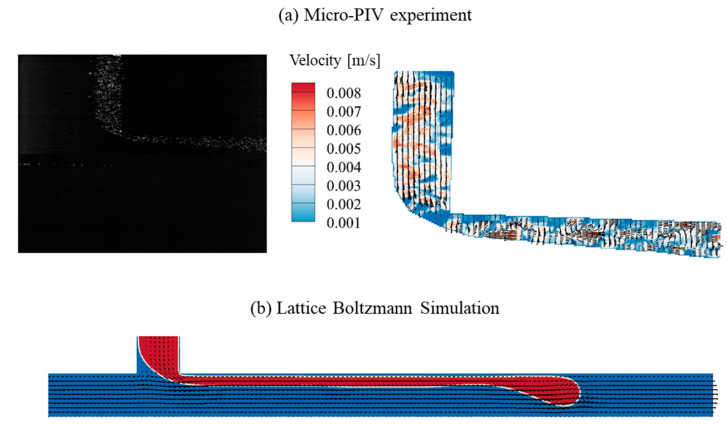
The interface and flow field obtained from the Micro-PIV experiment and the lattice Boltzmann simulation in the parallel regime with breakup pattern (*Q_C_* = 1.0 μL/min, *Q_D_* = 2.5 μL/min).

For the parallel regime with a breakup pattern (*Q_C_* = 1.0 μL/min, *Q_D_* = 2.5 μL/min), Figure 7 visualizes the interface and flow field obtained from the Micro-PIV experiment and lattice Boltzmann simulation. The corresponding dimensionless numbers are *Re_C_* = 0.843, *Ca_C_* = 2.11 × 10^−4^, *Re_D_* = 0.693, and *Ca_D_* = 1.91 × 10^−2^, respectively. While microdroplet generation is observed in both experimental and numerical results, the underlying mechanisms exhibit slight discrepancies, warranting further comparative analysis. In the present lattice Boltzmann simulation, the wetting boundary Equations (18) and (19) approaches the wettability of the solid surface using the relation between interfacial geometry and order parameter, but neglects the surface roughness effect, which may contribute to the observed discrepancies. Due to the absence of the surface roughness effect, a smooth velocity profile is observed in the numerical simulation, and the interface shape shows a similar deformation process to that in the threading regime. Moreover, 2 μm fluorescent tracing microparticles are introduced in the Micro-PIV experiments to visualize and track the flow field. These particles have an impact on the flow dynamics, particularly at higher flow rates. However, the effect of these tracing microparticles is neglected in the lattice Boltzmann simulations, which introduces another contributing factor to the discrepancies between the simulated and experimental velocity fields. Consequently, the velocity field obtained in the Micro-PIV experiment exhibits a more chaotic behavior, primarily due to both the effects of microchannel wall friction and fluorescent tracing microparticles. Furthermore, the unbalanced hydrodynamic forces of DI water and HFE-7100 compromise the interface stability. Consequently, the breakup position does not remain stationary but shifts dynamically due to the instability.

### 3.3. Quantitative Characterization of Microdroplet Properties

**Figure 8:**
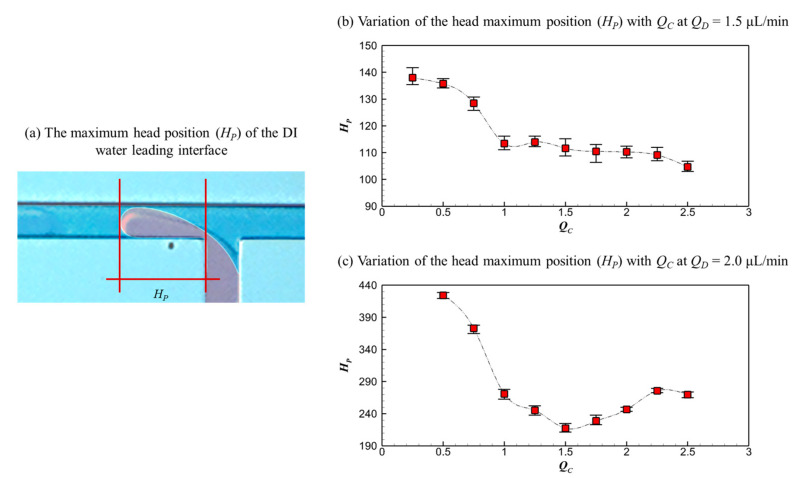
The maximum head position (*H_P_*) of the DI water leading interface at the farthest advancement: (**a**) schematic diagram, (**b**) *H_P_* varies with *Q_C_* at *Q_D_* = 1.5 μL/min, and (**c**) *H_P_* varies with *Q_C_* at *Q_D_* = 2.0 μL/min.

**Figure 9:**
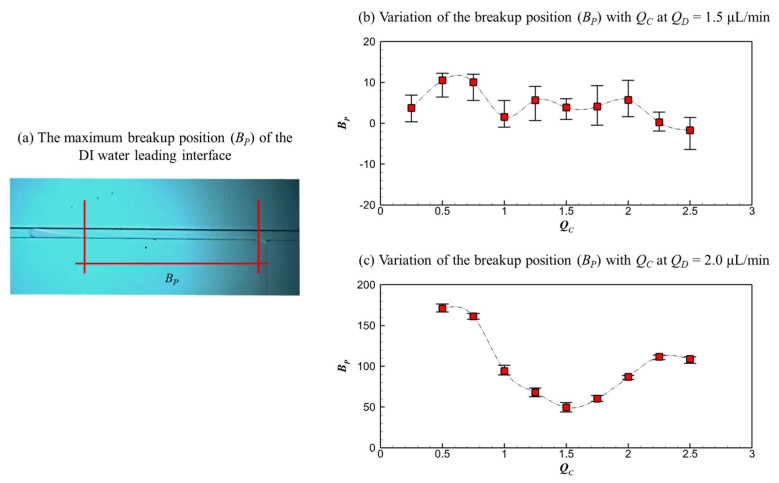
The breakup position (*B_P_*): (**a**) schematic diagram, varies with *Q_C_* at (**b**) *Q_D_* = 1.5 μL/min and (**c**) *Q_D_* = 2.0 μL/min.

To quantitatively characterize the microdroplet generation process, the maximum head position (*H_P_*) of the DI water leading interface and the breakup position (*B_P_*) are measured and presented in Figure 8 and Figure 9, respectively. All experiments are repeated at least 10 times, and the average values reported here are derived from a minimum of 10 valid measurements to ensure statistical reliability. A schematic diagram of *H_P_* is shown in Figure 8a, depicting the farthest advancement of the interface. *H_P_* in the dripping and threading regimes remains stationary, while that in the parallel regime with the breakup pattern moves dynamically due to the friction-induced interface instability. Figure 8b,c show *H_P_* in the dripping and threading regimes, respectively.

For the dripping regime (*Q_D_* = 1.5 μL/min), as shown in Figure 8b, the *H_P_* decreases with increasing *Q_C_*. As the flow rate of continuous fluids increases, the combined squeezing and shearing forces exerted by the continuous HFE-7100 flow on the dispersed DI water flow intensify, driving the reduction in head position. For the threading regime (*Q_D_* = 2.0 μL/min), as shown in Figure 8c, the *H_P_* first decreases and then increases with the increasing *Q_C_*. When *Q_C_* < 1.5 μL/min, the shearing effect of continuous HFE-7100 flow on the dispersed DI water flow increases with *Q_C_*, causing the breakup position to move closer gradually. When *Q_C_* > 1.5 μL/min, the gooseneck-like thin layer thins, and the head structure deforms under the competition between the surface tension effect of dispersed DI water and the squeezing effect of the continuous HFE-7100 flow, leading to the subsequent increase in *H_P_*.

Figure 9b,c show the variations of breakup position (*B_P_*) with *Q_C_* in the dripping and threading regimes, respectively. In the dripping regime, *B_P_* varies slightly with *Q_C_*, spanning from −1.70 μm to 10.51 μm, indicating stable droplets generated in this stage. The sign of *B_P_* denotes the direction of the reference coordinate system, as shown in Figure 9a, where positive values indicate the downstream direction and negative values indicate the upstream direction relative to the channel junction. In the threading regime, *B_P_* first decreases and then increases with increasing *Q_C_*, a trend that mirrors the non-monotonic variation of *H_P_* shown in Figure 8c. This correlation highlights the competition between the surface tension effect of the dispersed DI water and the squeezing effect of the continuous HFE-7100 flow, driving the coherent evolution of interface positions.

**Figure 10:**
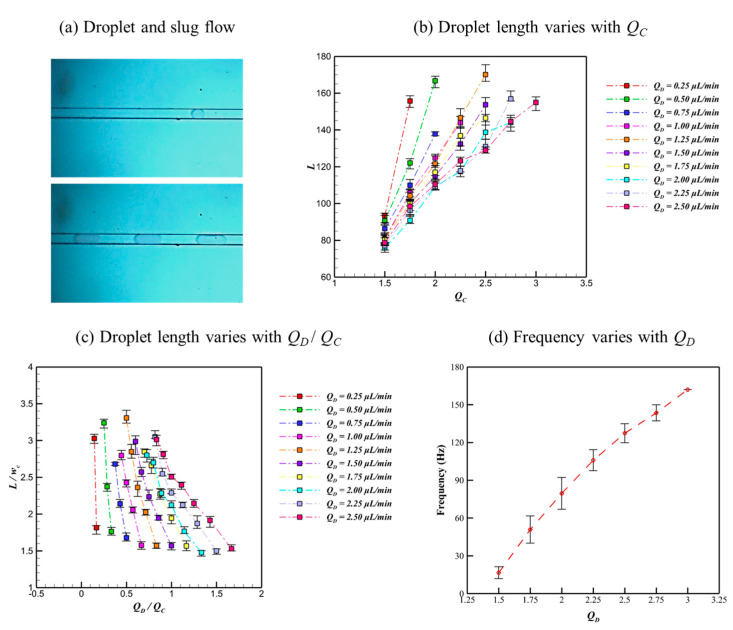
The properties of microdroplets generated in the T-junction microchannel: (**a**) droplet flow pattern, (**b**) droplet length varies with *Q_C_*, (**c**) dimensionless droplet length varies with the ratio of flow rate *Q_D_* / *Q_C_*, and (**d**) frequency varies with *Q_D_*.

Following the breakup of the dispersed DI water in the T-junction microchannel, a microdroplet forms and flows downstream toward the outlet, as shown in Figure 10a. At relatively low flow rates of both dispersed and continuous fluid, the generated microdroplets are small, sustaining a steady droplet flow pattern in the microchannel. Conversely, increasing the flow rate of either dispersed or continuous fluids triggers a transition to slug flow as the larger microdroplet forms due to increased dispersed fluid volume, or the breaking point moves downstream.

To quantitatively describe the microdroplet size, the droplet length (*L*) is measured and plotted as a function of *Q_C_* in Figure 10b. The results show that *L* increases with *Q_C_* but decreases with *Q_D_*, which reflects a competition between the squeezing and shearing effects of continuous fluid and the hydrodynamic and surface tension effects of dispersed fluid. With an increase in *Q_C_*, the squeezing force from the HFE-7100 flow acts on the DI water interface, elongating the gooseneck-like thin layer region and causing an increase in microdroplet volume. Conversely, with an increase in *Q_D_*, the hydrodynamic force of DI water flow becomes more prominent, which resists the growth of the gooseneck-like thin layer region. This interaction highlights the complex competition relation between the squeezing and shearing forces of the continuous fluid and the hydrodynamic and surface tension forces of the dispersed fluid, which collectively govern microdroplet formation, growth, and morphological evolution in the T-junction microchannel.

Furthermore, the dimensionless droplet length, defined as the ratio of droplet length *L* to microchannel width *w_c_*, exhibits a correlation with the flow rate ratio *Q_D_*/*Q_C_*, as depicted in Figure 10c. It shows that the dimensionless droplet length decreases rapidly with increasing *Q_D_*/*Q_C_*, and the slope of this correlation also decreases with increasing *Q_D_*. Moreover, the flow rate of dispersed fluid also has an impact on the frequency of the microdroplet generation, which is shown in Figure 10d. The results show that the frequency monotonically increases with *Q_D_*, and the maximum value approximates 162 microdroplets per second. Therefore, with an increase in *Q_D_*, the microdroplet with a smaller size is generated in the T-junction microchannel with higher efficiency, and the flow regime and microdroplet size could be further modulated by *Q_C_*.

## 4. Conclusions

In this study, the passive microdroplet generation process in a T-junction microchannel is systematically investigated using microscopic observations, Micro-PIV visualizations, and lattice Boltzmann simulations. The key flow regimes, i.e., dripping, threading, and parallel flow, are characterized by analyzing the balance between hydrodynamic forces and surface tension, revealing that the flow rate ratio of continuous fluid to dispersed fluid is a critical parameter governing regime transition. Micro-PIV visualizes the velocity field and vortex structures during droplet formation, while a lattice Boltzmann model with wetting boundary conditions predicts the interface deformation and flow dynamics. The lattice Boltzmann simulations exhibit good consistency with the experiments in the dripping and threading regimes, while showing discrepancies in the parallel flow regime due to the neglect of surface roughness and tracing microparticle effects at higher flow rates.

The present experimental results show that the maximum head position (HP) and breakup position (BP) of the dispersed phase exhibit non-monotonic trends when varying flow rates of continuous fluid in the threading regime, reflecting the competition between the squeezing and shearing forces of continuous fluid and the hydrodynamic and surface tension forces of dispersed fluid. Quantitative analysis reveals that droplet length increases with the flow rate of continuous fluid but decreases with the flow rate of dispersed fluid, while generation frequency monotonically increases with the flow rate of dispersed fluid, reaching a maximum of approximately 162 microdroplets per second.

The combined experimental and numerical approach highlights the importance of the Reynolds and capillary numbers in determining flow regimes and droplet characteristics. The lattice Boltzmann model effectively captures interface dynamics and validates the role of wetting properties in microchannel flows. Regarding the mechanism of microdroplet generation in the T-junction microchannel, as the *Q_D_* increases, the microdroplets are generated in a smaller size with higher efficiency. The flow regime and microdroplet size can be further modulated by *Q_C_*. This tunable relationship between flow rates and droplet characteristics holds great potential for advancing microfluidic applications in the fields of precision biology, advanced material fabrication, and targeted drug delivery, where precise manipulation of monodisperse droplets is essential for process optimization and performance improvement. Future research will focus on investigating the effects of microchannel geometries and external physical fields on the microdroplet generation process.

## Figures and Tables

**Table 1:** The physical properties of the test liquids.

Type	Material	Density (*ρ*)	Viscosity [56] (*μ*)	Surface Tension (*σ*)
Dispersed fluid	DI water	${998.20\;\mathrm{kg}/m}^{3}$	$1.0\;\mathrm{mN}\cdot {s/m}^{2}$	0.728 mN/m
Continuous fluid	HFE-7100	${1418.14\;\mathrm{kg}/m}^{3}$	$467.42\;\mu N\cdot {s/m}^{2}$	12.33 mN/m

## Data Availability

The data presented in this study are available on request from the corresponding author.
